# Metabolomics Analysis of Aged Garlic Extract for the Identification of Novel Compounds

**DOI:** 10.3390/metabo16060369

**Published:** 2026-05-29

**Authors:** Masato Nakamoto, Tsubasa Nishimura, Masahiro Ohtani, Toshiaki Matsutomo

**Affiliations:** 1 Central Research Institute, Wakunaga Pharmaceutical Co., Ltd., 1624 Shimokotachi, Koda-cho, Akitakata-shi 739-1195, Hiroshima, Japan; nishimura_t@wakunaga.co.jp (T.N.); ootani_m@wakunaga.co.jp (M.O.); matsutomo_t@wakunaga.co.jp (T.M.); 2 Graduate School of Integrated Sciences for Life, Hiroshima University, Higashi-Hiroshima-shi 739-8528, Hiroshima, Japan

**Keywords:** garlic, aged garlic extract, metabolomics analysis, identification

## Abstract

Background/Objectives: Aged garlic extract (AGE), produced by aging raw garlic in an aqueous ethanol solution for over 10 months, exhibits multiple pharmacological activities, including antioxidant and anti-inflammatory effects. However, because AGE has a complex composition and many constituents remain insufficiently characterized, the chemical basis underlying its broad activities is not fully understood. This study aimed to investigate these previously overlooked compounds in AGE to better understand its chemical complexity. Methods: AGE was fractionated using bioactivity assays to select target fractions for detailed chemical analysis. Metabolomics profiling was performed using liquid chromatography-mass spectrometry (LC-MS). Compounds were tentatively identified through database matching, fragmentation pattern analysis, and comparison with authentic standards. Results: Thirteen compounds not previously reported in AGE were tentatively identified. Citric acid was present at high levels. Citrulline and galacturonic acid were detected in AGE but not in raw garlic, suggesting that they are formed during the aging process. Trigonelline was detected and tentatively identified in the AGE sample used in this study. The remaining compounds included choline, 5-oxoproline, malic acid, gluconic acid, adenine, succinic acid, mucic acid, pipecolinic acid, and caffeic acid. These compounds may contribute to the diverse biological activities of AGE. Conclusions: These findings expand the chemical characterization of AGE and provide a foundation for understanding its broad pharmacological activities. They may also support future studies on functional food development and the health benefits of AGE.

## 1. Introduction

Garlic, one of the *Allium* plants, has been reported to exhibit various beneficial pharmacological effects [1]. To enhance the pharmacological activity of garlic and also reduce its characteristic pungent odor and irritation, several processed garlic preparations such as aged garlic extract (AGE) and black garlic have been developed. AGE is a unique garlic extract that is prepared by aging raw garlic in aqueous ethanol solution for more than 10 months [2]. Numerous studies have shown that AGE has diverse pharmacological effects such as enhancement of endurance capacity, anti-periodontal activity, attenuation of exercise-induced fatigue, and antioxidation and anti-inflammation activities [3,4,5,6]. To date, considerable research has been done on specific sulfur-containing amino acids (*S*-allylcysteine (SAC), *S*-1-propenylcysteine (S1PC) and *S*-allylmercaptocysteine (SAMC)) as the major contributors to the biological effects of AGE. These sulfur compounds have a wide range of pharmacological effects, including peripheral-circulation-improving, anti-inflammatory, anti-hypertensive, anti-atherosclerotic, and hepatoprotective effects [7,8,9,10,11]. Previous reports have demonstrated that these sulfur-containing amino acids play an important role in the beneficial actions of AGE. However, the composition of AGE is complex due to the presence of numerous components other than the aforementioned sulfur-containing amino acids [2]. In fact, few studies have examined other components in AGE beyond sulfur compounds, leaving other constituents insufficiently characterized. Therefore, further characterization of these less extensively studied AGE constituents is important for obtaining a more comprehensive understanding of the chemical profile of AGE and for providing a basis for future biological evaluation of its diverse pharmacological effects.

Bioactivity-guided screening has long been employed as an effective strategy for identifying biologically active constituents in complex plant extracts. By combining biological activity screening with analytical fractionation, it may be possible to narrow down the fractions containing components associated with specific biological effects [12,13]. This approach is particularly valuable for the research on natural products containing many compounds because it allows us to focus on biologically relevant fractions.

Metabolomics analysis integrates high-sensitivity analytical technologies to enable a non-targeted and simultaneous profiling of hundreds to thousands of metabolites in analyzed samples [14]. Given the highly complex and diverse chemical compositions of natural products, metabolomic studies predominantly employ analytical platforms such as liquid chromatography-mass spectrometry (LC-MS) and gas chromatography-mass spectrometry to detect a broad spectrum of compounds with varying chemical properties [15]. A key advantage of metabolomics analysis lies in its ability to detect minor constituents that are often overlooked by conventional targeted analytical approaches. By enabling comprehensive and unbiased profiling of extract components, this methodology substantially expands the potential for discovering novel or previously uncharacterized compounds. Therefore, metabolomics analysis represents an effective and complementary approach for the non-targeted, comprehensive investigation of plant extract constituents.

In this study, we applied an integrated strategy combing bioactivity-guided screening with metabolomics analysis to investigate the chemical constituents of AGE. Bioactivity-guided screening based on anti-inflammatory activity was employed as an initial guide for fractionation to narrow down the analytical targets, while subsequent metabolomics profiling was used to comprehensively characterize the constituents present in the selected fractions. A bioactivity-guided assay was conducted using inhibition of interleukin-6 (IL-6) production, a reported biological activity of AGE [16]. We hypothesized that, although the complex and insufficiently characterized composition of AGE makes it difficult to fully understand its broad pharmacological effects, integrating bioactivity-guided fractionation with metabolomics analysis would enable the characterization of previously unidentified constituents of AGE and provide a more comprehensive approach to profiling its bioactive components. Through this approach, we detected and tentatively identified 13 compounds of AGE including citric acid, galacturonic acid, citrulline, and trigonelline.

## 2. Materials and Methods

### 2.1. Chemicals and Reagents

Garlic bulbs (*Allium sativum*) were obtained from Wakunaga of America (Madero, CA, USA). AGE was manufactured by aging sliced garlic in aqueous ethanol over 10 months at room temperature [United States Pharmacopeial Convention I, United States Pharmacopoeia 38Garlic Fluidextract USP 38–NF 33, United States Pharmacopeial Convention, Rockville, MD, 2015, pp. 6052–6055]. Formic acid (FA) with LC-MS grade was purchased from FUJIFILM Wako Pure Chemical Corporation (Osaka, Japan). Methanol and acetonitrile with HPLC grade were from Kanto Chemical (Tokyo, Japan). Deionized water was purified by a Milli-Q system (Millipore, Tokyo, Japan). D-α-galacturonic acid (purity > 95.0%), L-pyroglutamic acid (L-5-oxoproline) (purity > 97.0%), L-(-)-malic acid (purity > 98.0%), pipecolinic acid (purity > 98.0%), and mucate (purity > 97.0%) were purchased from Tokyo Chemical Industry (Tokyo, Japan). Choline chloride (purity > 95%), 50% gluconic acid solution, L-citrulline (purity > 98.0%), citric acid (purity > 98%), succinic acid (purity > 99.5%), and adenine (purity > 95.0%) were from FUJIFILM Wako Pure Chemical Corporation. An amino acid mixture standard solution (Type H) (L-cystine: 0.13 μmol/mL, other amino acids: 0.25 μmol/mL) was purchased from FUJIFILM Wako Pure Chemical Corporation. Trigonelline (purity > 95.0%) was from Toronto Research Chemicals (Toronto, ON, Canada). Caffeic acid (purity > 98.0%) was purchased from Sigma-Aldrich (St. Louis, MO, USA). Authentic sulfur compound for identification were synthesized and purified according to previous reports [17].

### 2.2. Characterization Methods of AGE

HPLC analysis was performed via the post-column HPLC method using a NEXERA X2 system (Shimadzu, Kyoto, Japan) according to a previous report [17]. The LC-MS analysis was carried out on a system consisting of an UltiMate 3000 (Dionex/Thermo Fisher Science, Waltham, MA, USA) coupled to a Q-Exactive (Thermo Fisher Scientific, Waltham, MA, USA) according to a previous report [17]. The column utilized for separation was a Cadenza CD-C18 column (2.0 mm × 150 mm, 3 μm, Imtakt Corporation, Kyoto, Japan).

### 2.3. IL-6 Production Assay

The IL-6 assay was carried out as described by Suzuki et al. [18]. Female C57BL/6N mice were obtained from CLEA Japan (Tokyo, Japan) and individually housed under specific pathogen-free condition. Those mice were provided with a commercial diet (CE-2; CLEA, Tokyo, Japan) and water ad libitum under a 12 h light–dark cycle, controlled temperature (23 ± 1 °C) and humidity (55 ± 5%). All animal procedures were in accordance with the protocol approved by the Wakunaga Pharmaceutical Company (Tokyo, Japan) Institutional Animal Care and Use Committee (No. 0367). After cervical dislocation, spleen dissected out from mice (8–13 weeks old) was homogenized with a 5 mL syringe (JMS, Tokyo, Japan). The homogenates were then filtered through a 40 µm cell strainer (Corning, Tokyo, Japan), followed by centrifugation at 4 °C for 5 min (1800 rpm). The red blood cells contaminated in the homogenates were lysed by adding lysing solution (BD Biosciences, Tokyo, Japan). After further centrifugation, lymphocytes obtained were seeded onto each well of 48-well plates at 1 × 10^6^ cells/mL in RPMI1640 (250 µL) supplemented with 10% FBS, 100 U/mL penicillin, and 100 µg/mL streptomycin (Wako, Osaka, Japan). After seeding, the cells were simultaneously treated with LPS (1 µg/mL) and AGE or its separated fractions (solid weight corresponding to 4 mg/mL of AGE) for 24 h. After treatment, the content of IL-6 in the supernatant was determined through enzyme-linked immunosorbent assay (ELISA) (Invitrogen, Carlsbad, CA, USA), according to the manufacturer’s protocol. All values below 40 pg/mL were considered zero, as they were below the lower limit of the standard curve. Independent sample preparations were performed 4–5 times using spleens obtained from multiple mice, and each independent preparation was considered a biological replicate. Technical replicates were performed for some assays and are indicated in the corresponding figure legends.

### 2.4. Cell Proliferation Assay

The lymphocytes isolated from mouse spleen were cultured in a 96-well plate at 1.0 × 10^5^ cells/mL, and then treated with LPS (1 µg/mL) in the absence (control) or AGE (4 mg/mL) for 24 h in RPMI medium containing 10% FBS, 100 U/mL penicillin and 100 µg/mL streptomycin. After treatment, bromodeoxyuridine (BrdU) reagent was added to the cells and cultured for further 24 h. The rate of incorporated BrdU into cells was assessed according to manufacturer’s protocol.

### 2.5. First Fractionation of AGE Through Dialysis

AGE (30 mL) was placed in a dialysis tube and dialyzed against 3000 mL of distilled water overnight with stirring. The outer solution was collected and another 3000 mL of distilled water was added to the beaker. After the same operation was repeated twice, the combined outer solution (Fr. 1) and inner solution (Fr. 2) were collected and lyophilized.

### 2.6. Second Fractionation of AGE Through Ion Exchange Chromatography

After Fr.1 (2 g) was placed in an Erlenmeyer flask and dissolved in water (300 mL), cation exchange resin (dowex 50Wx8, DowDuPont Inc., Wilmington, DE, USA) (10 mL) was added. The resultant mixture was slowly transferred into a chromatogram tube. Compounds retained on the column were eluted with 2 M ammonia water, which was allowed to flow until the eluate was neutral. Both the bare solution (Fr.1–1) and 2 M ammonia water eluate (Fr.1–2) were concentrated and dried using an evaporator and lyophilization.

### 2.7. Third Fractionation of AGE Through High-Performance Liquid Chromatography (HPLC)

Fr.1–1 was fractionated by a preparative HPLC (Nihon bunko, Tokyo, Japan). An aliquot (1 mL) of Fr.1–1 solution dissolved in water (2 mg/mL) was injected onto a Cadenza C18 column (250 mm × 10 mm, 5 μm, Imtakt, Kyoto, Japan) equipped with a Cadenza C18 guard column at 40 °C. The mobile phase consisted of solvent A (water) and solvent B (methanol) at a flow rate of 2.5 mL/min, with the gradient elution as follows: 0–15 min, 5% B; 15–40 min, 5–80% B; 40–40.1 min, 80–100% B; 40.1–50 min, 100% B; 50–56 min, 100–5% B; and 56–66 min, 5% B. Eleven fractions obtained were named Fr.1–1–1 (0–6 min), Fr.1–1–2 (6–12 min), Fr.1–1–3 (12–18 min), Fr.1–1–4 (18–24 min), Fr.1–1–5 (24–30 min), Fr.1–1–6 (30–36 min), Fr.1–1–7 (36–42 min), Fr.1–1–8 (42–48 min), Fr.1–1–9 (48–54 min), Fr.1–1–10 (54–60 min), and Fr.1–1–11 (60–66 min). Each fraction was freeze-dried and stored until further analysis.

### 2.8. Sample Preparation for Metabolomics

An aliquot of Fr.1–1 sample (50 μL) was mixed with both internal standard (IS, *S*-Butenylcysteine) (synthesized in-house) solution (50 μL of 0.05 mg/mL IS in 20 mM HCl) and 50% methanol (400 μL). The mixture was shaken vigorously for 0.5 min, filtrated through a 0.22 μm membrane, and analyzed using LC-MS.

### 2.9. Metabolomics Analysis Methods

The fraction samples were analyzed by means of UltiMate3000 UHPLC systems (Thermo Fisher Scientific, Yokohama, Japan) coupled with a Q-Exactive quadrupole-Orbitrap mass spectrometer (Thermo Fisher Scientific, Yokohama, Japan). An aliquot (10 μL) of each sample was injected onto a Cadenza C18 column (150 mm × 2.0 mm, 3 μm, Imtakt, Kyoto, Japan) equipped with a Cadenza C18 guard column at 40 °C. The mobile phase consisted of (A) 0.1% FA in water and (B) 0.1% FA in 80% methanol at a flow rate of 0.2 mL/min, with the gradient elution as follows: 0–9 min, 0% B; 9–12 min, 0–40% B; 12–19 min, 40% B; 19–22 min, 40–100% B; 22–25 min, 100% B; 25–25.01 min, 100–0% B; and 25.01–32 min, 0% B. Mass spectrometry was operated with an electrospray ionization (ESI) source in positive and negative-ion mode. For data acquisition of the components, the eluent was analyzed using full scan mode at a resolution of 70,000 at *m*/*z* 200. The operation parameters were as follows: vaporizer temperature of ESI probe, 300 °C; spray voltage, 4.5 kV (ESI^+^) or −3.5 kV (ESI^−^); capillary temperature, 350 °C; sheath gas, 40 arbitrary units; and auxiliary gas, 10 arbitrary units. The scan range was set to *m*/*z* 200–2000. In addition, the eluent was analyzed using dd-MS2 (Top 10) mode at a resolution of 17,500 at *m*/*z* 200. The operation parameters were as follows: vaporizer temperature of ESI probe, 300 °C; spray voltage, 4.0 kV (ESI^+^) or −3.5 kV (ESI^−^); capillary temperature, 250 °C; sheath gas, 50 arbitrary units; auxiliary gas, 10 arbitrary units; and collision energy, 20, 40, and 60. The scan range was set to *m*/*z* 58–870. Although a full analytical validation was not performed in this study, the mass spectrometer was calibrated prior to analysis to ensure mass accuracy. Peak assignment was based on the measured accurate mass and retention behavior under the LC-MS conditions used in this study.

### 2.10. Quantification of AGE Compounds

AGE and its fractionated samples were analyzed by means of UltiMate 3000 UHPLC systems (Thermo Fisher Scientific, Yokohama, Japan) coupled with a Q-Exactive quadrupole-Orbitrap mass spectrometer (Thermo Fisher Scientific, Yokohama, Japan). An aliquot (1 μL) of each sample was injected onto a Cadenza C18 column (150 mm × 2.1 mm, 3 μm, Imtakt, Kyoto, Japan) equipped with a Cadenza C18 guard column at 40 °C. Gradient conditions and other parameters were measured under the same LC-MS conditions as those used for the metabolomics analysis. In addition, for the identification and quantification of AGE compounds, parallel reaction monitoring mode was performed at a resolution of 17,500 at *m*/*z* 200 with optimal collision energy. The contents of the identified compounds were estimated in mg/g dry weight using calibration curves with an IS. These values were used for comparative purposes. As full method validation was not performed, the values should be regarded as approximate.

### 2.11. Data Processing

The raw LC-MS data were converted using the Analysis Base File Converter (Reifycs Inc., Tokyo, Japan). The peak detection and integration were performed on the MassBank datasets, (https://massbank.jp/MassBank/; MSMS-Neg-MassBank, accessed on 19 May 2025; MSMS-Pos-MassBank, accessed on 22 May 2025) derived from full scan analysis using MS-DIAL ver 4.90 software (RIKEN Center for Sustainable Resource Science: Metabolome Informatics Research Team, Yokohama, Japan). Missing values corresponding to undetected compounds were interpreted as indicating that the respective compounds were either absent from the sample or present at concentrations below the limit of detection. Candidate compounds were identified through a three-step process as follows (Figure S1): Process 1: compounds whose MS/MS spectra matched entries in the MassBank database were extracted and tentatively identified. Process 2: from these, only compounds with peaks detected in AGE were selected. Process 3: their retention times and fragment ions were confirmed using authentic standards.

### 2.12. Statistical Analysis

All data were first assessed for normality and homogeneity of variance before selecting the appropriate statistical tests. Normality was evaluated using the Shapiro-Wilk test, and homogeneity of variance was evaluated using Levene’s test. For datasets that satisfied both normality and homogeneity of variance, one-way analysis of variance (ANOVA) followed by Tukey’s post hoc test was used. For datasets that satisfied normality but not homogeneity of variance, Welch’s ANOVA followed by the Games-Howell test was used. For datasets that did not satisfy normality and homogeneity of variance, the Kruskal-Wallis test followed by Steel’s test was used. Grubb’s test was used to exclude outliers judged as *p* values lower than 0.05 in the datasets. Statistically significant difference was considered as *p* < 0.05. All statistical analyses were performed using EZR (Saitama Medical Center, Jichi Medical University, Saitama, Japan) [19].

## 3. Results

### 3.1. Characterization of AGE

AGE was characterized through post-column HPLC method using hexaiodoplatinate reagent which used for sulfur-specific detection [17]. According to the analysis, 8 hydrophilic sulfur compounds were identified in AGE (Figure S2 and Table S1). Some of them were produced through the aging process and these compounds, such as *S*-methylcysteine, SAC, S1PC, and SAMC, are characteristic sulfur compounds in aged garlic extract [2,17].

### 3.2. Determination of Sample Concentrations for Assay

We first examined the activity of AGE to inhibit IL-6 production in mouse splenic lymphocytes stimulated with LPS in vitro. The effect of AGE on LPS-induced IL-6 production was evaluated at concentrations of 0.04, 0.4, and 4 mg/mL. AGE inhibited LPS-induced IL-6 production in a concentration-dependent manner, with an estimated IC_50_ value of 0.52 mg/mL (Figure S3a). In contrast, AGE did not affect cell proliferation or viability even at the highest concentration of 4 mg/mL (Figure S3b). We found that AGE inhibited LPS-induced IL-6 production in a concentration-dependent manner (Figure S3a), without affecting cell proliferation and viability even at the highest concentration of 4 mg/mL (Figure S3b). Based on these findings, subsequent bioassays of AGE fractions were performed at a concentration equivalent to 4 mg/mL of AGE to allow a direct comparison of the activity contribution of AGE in each fraction.

### 3.3. Fractionation of AGE

Since AGE is a mixture containing a variety of substances including sugars (polysaccharides and monosaccharides), proteins, and low-molecular-weight components (e.g., amino acids), identification of the ingredients in AGE requires a carefully designed fractionation strategy [2]. The most commonly used methods for fractionation of extracts derived from animal or plant are liquid–liquid extraction, column chromatography, and HPLC [20]. Liquid–liquid extraction and column chromatography are effective techniques for the coarse separation of extracted components into broad classes, whereas HPLC is effective for the fine fractionation of complex mixture. Liquid–liquid extraction was deemed unsuitable for fractionation of AGE, since AGE was prepared by extracting raw garlic using aqueous ethanol, rendering partitioning in organic solvent systems impractical. Moreover, although AGE contains polysaccharides and proteins, column chromatography and HPLC generally have limited applicability for the efficient isolation and recovery of high-molecular-weight components. Thus, we chose a multi-step fractionation combining dialysis, column chromatography using ion exchange resins, and HPLC in order to achieve effective separation and recovery of the ingredients contained in AGE without any loss.

**Figure 1:**
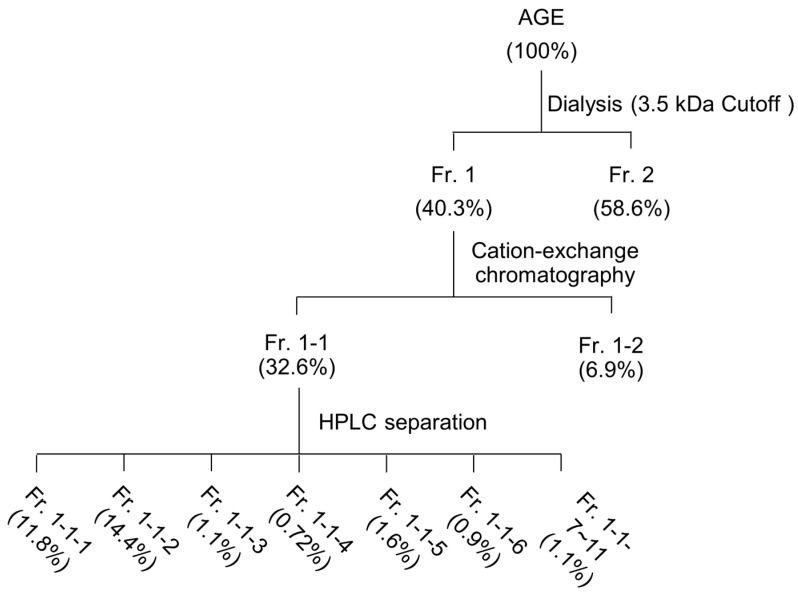
Fractionation scheme of AGE. At the first step, AGE was fractionated by dialysis to yield Fr.1 (low-molecular-weight fraction) and Fr.2 (high-molecular-weight fraction). At the second step, Fr.1 was fractionated by cation exchange chromatography to yield Fr.1–1 and Fr.1–2. At the third step, Fr.1–1 was fractionated by HPLC to yield 7 fractions (Fr.1–1–1~Fr.1–1–6 and Fr.1–1–7~Fr.1–1–11). The percentages in Figure 1 indicate the recovery amount when total solid weight in AGE is set to 100%.

**Figure 2:**
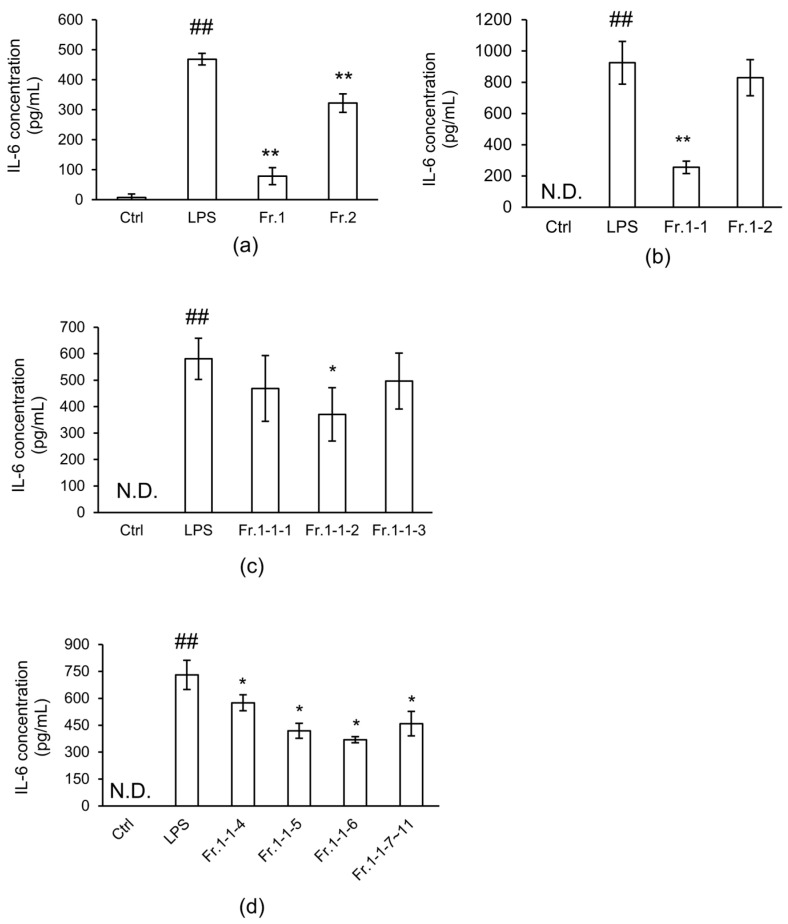
Effect of AGE fractions on IL-6 production in mouse splenic lymphocytes. (**a**–**d**) Splenic lymphocytes were untreated (control, ctrl) or treated with 1 µg/mL lipopolysaccharide (LPS) alone, LPS plus (**a**) Fr.1 or Fr. 2, (**b**) Fr.1–1 or –2, (**c**) Fr.1–1–1, –2 or –3, (**d**) Fr. 1–1–4, –5, –6 or –7~11 (solid weight corresponding to 4 mg/mL of AGE) for 24 h. The content of IL-6 in the culture medium was determined through ELISA. Values are mean ± SD (*n* = 4–5). Experiments were performed independently twice (**a**) and (**b**), once (**c**) and (**d**) as biological replicates, and each experiment included 4–5 technical replicates. ## *p* < 0.01 vs. control, * *p* < 0.05 vs. LPS alone, ** *p* < 0.01 vs. LPS alone. Normality and homogeneity of variance were assessed using the Shapiro–Wilk and Levene’s tests, respectively. Statistical comparisons were performed using one-way ANOVA with Tukey’s post hoc test, Welch’s ANOVA with the Games-Howell test, or the Kruskal-Wallis test with Steel’s post hoc test, as appropriate. N.D. means not detected.

First, molecular weight-based separation through dialysis yielded the low-molecular-weight fraction (Fr. 1, 40.3%) and the high-molecular-weight fraction (Fr. 2, 58.6%) from AGE (100%) (Figure 1). We found that both Fr.1 and Fr.2 exhibited significant inhibitory effects on LPS-stimulated IL-6 production, while the effect of Fr.2 was considerably weak compared with that of Fr.1 (Figure 2a). Fr.2 was rich in garlic-derived fructans which have been reported to suppress LPS-induced IL-6 production in RAW 264.7 macrophage cells [21].

Since Fr.1 showed a greater activity than Fr.2, this fraction was further fractionated by cation exchange chromatography into Fr.1–1 (32.6%) and Fr.1–2 (6.9%) with high recovery rates (Figure 1). Fr.1–1 showed the robust inhibitory effect on LPS-stimulated IL-6 production, whereas Fr.1–2 had no effect (Figure 2b). This result suggested that key ingredients responsible for the IL-6 inhibitory activity of AGE were present in Fr.1–1. Subsequent fractionation of Fr.1–1 was performed through HPLC yielding 11 subfractions (Figure 1). Among those subfractions, Fr.1–1–2, Fr.1–1–4, Fr.1–1–5, Fr.1–1–6, and Fr.1–1–7~11 (mixture of five subfractions) inhibited the LPS-stimulated IL-6 production (Figure 2c,d). The dispersion of bioactivity across multiple fractions made it difficult to reduce the number of fractions subjected to detailed analysis, which was the primary purpose of the screening in this study. Consequently, we proceeded with a comprehensive analysis focusing on Fr.1–1.

### 3.4. Metabolomics Analysis

**Figure 3:**
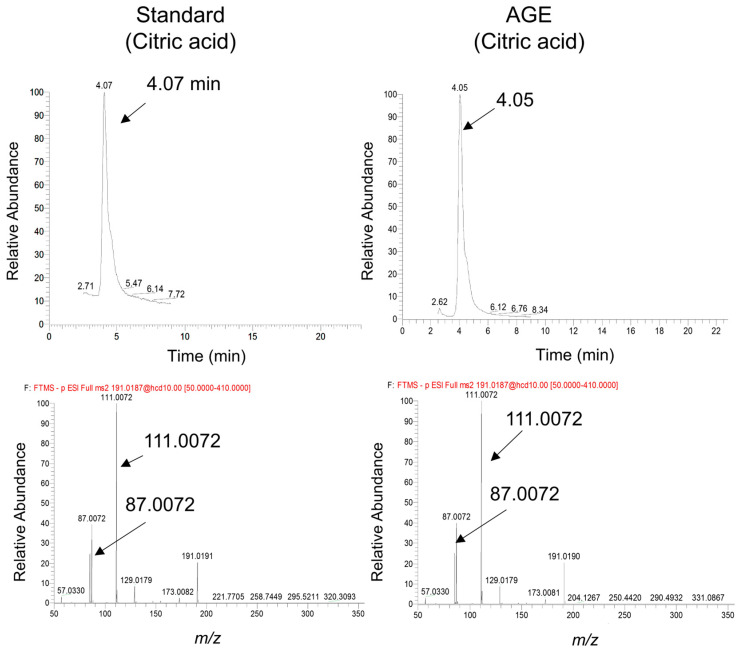
Tentative identification of the compound through LC-MS/MS. The top and bottom figures show the LC-MS chromatogram and MS/MS spectrum, respectively.

**Figure 4:**
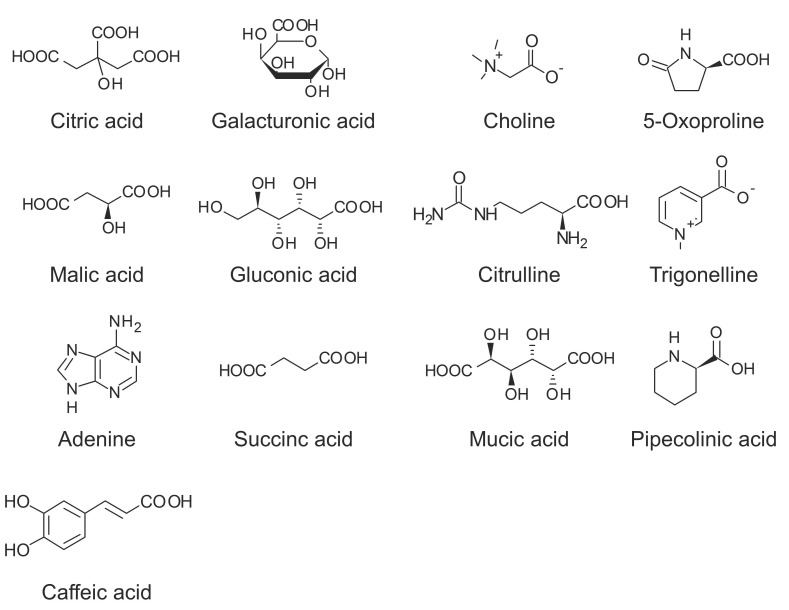
The compounds tentatively identified in AGE for the first time.

Using this strategy, we thoroughly analyzed the chemical constituents of Fr.1–1 consisting of all subfractions (Fr.1–1–1~11). Comprehensive metabolomic profiling enabled the tentative annotation of a broad range of compounds. Following stringent peak-quality assessment in AGE, 64 compounds exhibiting clearly defined and reliable signals were selected for further analysis. Among these, 31 compounds, including standard amino acids, were extracted using a peak-intensity cutoff value of 1.0 × 10^6^. Public databases generally provide MS/MS data without associated retention time information, which can result in false-positive hits when compounds share identical masses. To address this problem, hit compounds present in AGE were validated by comparing their retention times with those of authentic standards using LC-MS (Thermo Fisher Scientific) (Figure S4a–m). As shown for citric acid in Figure 3, each compound was identified by comparing the retention time, precursor ion (*m*/*z* 191), and fragmentation pattern (*m*/*z* 191 > 111 and 87) between the authentic standard and the sample. Through this process, 25 compounds belonging to diverse chemical classes, including sugar acids, amino acids, and phenolic acids, were tentatively identified (Table 1 and Figure 4). Although no IL-6 production-inhibitory activity was observed for these compounds in the present assay, 13 constituents were tentatively identified in AGE for the first time. Subsequently, the contents of the identified compounds in AGE were quantitatively analyzed using authentic standards. In addition, to evaluate the potential influence of aging process of AGE on constituent composition, the presence of the tentatively identified compounds was also examined in fresh garlic. The quantitative values in both AGE and raw garlic are shown in Table 1.

## 4. Discussion

Metabolomics analysis is a widely used method for characterizing metabolic variations and comprehensively profiling sample constituents without restricting the analysis to predefined targets. For example, Lee et al. applied untargeted metabolomics to systematically characterize antioxidant-related metabolites in Betulaceae plant extracts, and Zheng et al. utilized untargeted metabolomics combined with bioassays to prioritize metabolites associated with antioxidant activity [22,23]. These studies support the usefulness of metabolomics-based component characterization. One major advantage of metabolomics analysis is its ability to minimize the risk of overlooking biologically active minor constituents by enabling holistic compound identification. In the present study, although none of the tentatively identified compounds exhibited IL-6 inhibitory activity, a total of 25 compounds were successfully identified in AGE using a combined workflow of bioactivity-guided screening and metabolomic analysis. The tentatively identified compounds exhibited a wide range of abundances from several µg/g dry weight to over 10 mg/g dry weight. In addition, several compounds, such as galacturonic acid and citrulline, which are present at low levels in raw garlic but increase in abundance in AGE, were found. These observations indicate that AGE contains a chemically diverse set of constituents.

Citric acid, an organic acid widely distributed in citrus plants [24], was detected at high concentrations in AGE. Citric acid has been reported to improve formulation properties in foods and other products, including inhibition of spoilage and masking of undesirable flavors [25,26]. In addition, citric acid may exert effects such as pH adjustment and inhibition of bacterial growth during the aging process used to prepare AGE. The presence of citric acid in AGE had not been previously reported to date. Some of the compounds identified in this study, including citric acid, are detectable only in the negative-ion mode during LC-MS analysis. Owing to such methodological limitation, accurate identification of these compounds was achieved by employing a comprehensive analytical strategy in this study, which fully utilized both positive and negative ionization modes.

Galacturonic acid is a monosaccharide compound constructing pectin, a major constituent of plant cell walls, and is a bioactive compound that has been reported to modulate immune function [27,28]. Pectin is broken down into galacturonic acid by pectinase within plants or acid hydrolysis [29]. In raw garlic, galacturonic acid exists predominantly in polymerized form as part of pectin and is therefore not detected as a free compound. However, galacturonic acid is generated during the aging process, likely through cleavage of glycosidic bonds mediated by pectinase and acid hydrolysis in the extract. Similar to citric acid, galacturonic acid was detectable only in the negative-ion mode, confirming its presence in AGE through comprehensive metabolomic analysis.

Citrulline is a non-proteinogenic amino acid commonly found in specific plant species, such as watermelon [30]. Notably, citrulline was not detected in raw garlic, and, based on the available literature, there have been no reports describing its presence in garlic-derived products. Citrulline is generally produced through arginine biosynthesis and its accumulation has been reported to be enhanced in response to environmental stress [31]. In addition, arginine-to-citrulline conversion has been reported to occur during alcohol fermentation in the process of soy sauce production, resulting in increased citrulline levels [32]. Although the precise formation pathway of citrulline in AGE remains unclear, the high arginine content in raw garlic and AGE suggests that its generation may be induced by the aging process under ethanol–aqueous environment used for AGE preparation.

In addition to the compounds discussed above, several other metabolites detected in the present study have previously been reported in garlic-derived materials other than AGE. For example, trigonelline and caffeic acid were reported in “Sulmona Red Garlic” aerial bulbils, and trigonelline has also been isolated from tissue cultures of *Allium sativum* [33]. Pipecolinic acid was detected in a garlic hydrophilic extract, and choline, 5-oxoproline, succinic acid, and malic acid have been described in the context of garlic bioactive substances and metabolites [34,35,36]. These previous reports indicate that some metabolites identified in the present study should not be regarded as entirely novel within garlic-derived products in general. However, their presence in AGE has not been well documented, and differences in garlic variety, sample type, processing conditions, aging process, extraction methods, and analytical platforms should be considered when comparing these findings. Therefore, the present study extends the existing literature by confirming these metabolites in AGE and integrating them into the AGE-specific chemical profile associated with the biological effects observed here.

However, none of the 13 compounds examined in this study showed IL-6 inhibitory activity at concentrations corresponding to those present in AGE. Thus, the IL-6 inhibitory activity of AGE cannot be attributed to any single compound evaluated under the tested conditions. This finding suggests that the observed activity may reflect the combined contribution of multiple constituents, including minor or unidentified compounds. Accordingly, the compounds identified in this study should be interpreted as part of the chemical characterization of AGE rather than as confirmed active constituents.

A limitation of this study is that compound identification relied on existing databases, and thus compounds not included in these databases could not be identified. In addition, the analytical approach used in this study was not sufficient to comprehensively evaluate volatile compounds, because the LC-MS platform was primarily suited for non-volatile or semi-polar metabolites. Therefore, volatile compounds that may contribute to the biological effects of AGE may have been overlooked. Future studies using expanded databases and complementary analytical approaches, such as gas chromatography-mass spectrometry, are required to further elucidate the unidentified chemical profile of AGE.

In the present study, we identified 13 compounds in AGE that have not been reported, by combining bioactivity-guided screening with metabolomic analysis. Among those, citric acid was found to be present at particularly high levels, while galacturonic acid and citrulline appeared to be generated during the aging process. Trigonelline was a new constituent not previously identified in garlic or garlic products. A diverse array of chemical classes, including sugar acids, amino acids, and organic acids, was identified in AGE. These constituents may contribute to the multifunctional properties of AGE reported to date.

## Figures and Tables

**Table 1:** Analytical data for compounds in AGE.

Identified Compounds	Retention Time	Ion Types	Theoretical *m*/*z*	Actual *m*/*z*	MS Fragment	Contents in AGE (mg/g Dry)	Level in AGE	Contents in Raw Garlic (mg/g Dry)	Level in Raw Garlic
**Citric acid**	**4.08**	**[M−H]^−^**	**191.0192**	**191.0190**	**111.0072**	**17.21**	**High**	**18.14**	**High**
Glutamic acid	1.73	[M+H]^+^	148.0610	148.0600	84.0450	12.22	High	3.084	Medium-high
Arginine	1.60	[M+H]^+^	175.1195	175.1185	70.0658	7.051	High	4.102	Medium-high
**Galacturonic acid**	**1.84**	**[M−H]^−^**	**193.0348**	**193.0341**	**72.9916**	**4.130**	**Medium-high**	**0.000**	**N.D.**
**Choline**	**1.71**	**[M]^+^**	**104.1075**	**104.1071**	**104.1071**	**2.324**	**Medium-high**	**0.879**	**Medium**
**5-oxoproline**	**4.25**	**[M−H]^−^**	**128.0348**	**128.0339**	**128.0339**	**1.454**	**Medium-high**	**0.303**	**Low**
**Malic acid**	**2.52**	**[M−H]^−^**	**133.0137**	**133.0129**	**115.0021**	**1.131**	**Medium-high**	**0.778**	**Medium**
**Gluconic acid**	**1.84**	**[M−H]^−^**	**195.0505**	**195.0504**	**129.0180**	**0.972**	**Medium**	**0.000**	**Trace**
Phenylalanine	12.98	[M+H]^+^	166.0868	166.0857	120.0810	0.903	Medium	0.331	Low
Proline	2.02	[M+H]^+^	116.0712	116.0707	70.0658	0.843	Medium	1.744	Medium-high
Aspartic acid	1.72	[M+H]^+^	134.0453	134.0448	88.0398	0.728	Medium	1.570	Medium-high
**Citrulline**	**1.81**	**[M+H]^+^**	**176.1035**	**176.1030**	**159.0763**	**0.704**	**Medium**	**0.000**	**Trace**
Leucine	5.70	[M+H]^+^	132.1025	132.1017	86.0969	0.482	Low	0.152	Low
Valine	2.60	[M+H]^+^	118.0868	118.0862	72.0814	0.465	Low	0.373	Low
Tyrosine	5.51	[M+H]^+^	182.0817	182.0807	136.0758	0.363	Low	0.630	Medium
Isoleucine	5.26	[M+H]^+^	132.1025	132.1017	86.0970	0.224	Low	0.108	Low
**Trigonelline**	**2.02**	**[M+H]^+^**	**138.0555**	**138.0550**	**94.0656**	**0.221**	**Low**	**0.117**	**Low**
**Adenine**	**2.71**	**[M+H]^+^**	**136.0623**	**136.0615**	**136.0618**	**0.137**	**Low**	**0.000**	**N.D.**
Threonine	1.74	[M+H]^+^	120.0661	120.0655	102.0554	0.251	Low	0.162	Low
Serine	1.70	[M+H]^+^	106.0504	106.0500	60.0451	0.203	Low	0.741	Medium
**Succinic acid**	**4.82**	**[M−H]^−^**	**117.0188**	**117.0179**	**73.0279**	**0.129**	**Low**	**0.024**	**Low**
Histidine	1.57	[M+H]^+^	156.0773	156.0767	110.0716	0.082	Low	0.328	Low
**Mucic acid**	**1.89**	**[M−H]^−^**	**209.0297**	**209.0278**	**133.0129**	**0.061**	**Low**	**0.133**	**Low**
**Pipecolinic acid**	**2.74**	**[M+H]^+^**	**130.0868**	**130.0862**	**84.0814**	**0.036**	**Low**	**0.065**	**Low**
**Caffeic acid**	**17.30**	**[M−H]^−^**	**179.0344**	**179.0339**	**135.0438**	**0.031**	**Low**	**0.000**	**Trace**

Bold entries indicate compounds newly identified in AGE. Compounds were categorized based on their content levels as follows: high (>5 mg/g dry), medium-high (1–5 mg/g dry), medium (0.5–1 mg/g dry), and low (<0.5 mg/g dry). N.D.: not detected. Trace: detected but below the quantification.

## Data Availability

The original contributions presented in this study are included in the article. Further inquiries can be directed to the corresponding author.

## Supplementary Materials

The following supporting information can be downloaded at: https://www.mdpi.com/article/10.3390/metabo16060369/s1, Figure S1: Identification process of the components in 3rd separated fractions of AGE; Figure S2: Effect of AGE on IL-6 production and cell viability in mouse splenic lymphocytes; Figure S3: Chromatogram of hydrophilic sulfur compounds in aged garlic extract by post-column HPLC analysis; Figure S4: (a)–(m). Tentative identification of the compound by LC-MS/MS; Table S1: Characterization of hydrophilic sulfur compound in AGE from post-column HPLC chromatogram.

## Abbreviations

The following abbreviations are used in this manuscript:

AGE	aged garlic extract
BrdU	bromodeoxyuridine
ELISA	enzyme-linked immunosorbent assay
HPLC	high-performance liquid chromatography
IL-6	interleukin-6
IS	internal standard
LPS	lipopolysaccharide
LC-MS	liquid chromatography–mass spectrometry
SAC	*S*-allylcysteine
SAMC	*S*-allylmercaptocysteine
S1PC	*S*-1-propenylcysteine
