# Supplementary material for: Metabolomics Analysis of Aged Garlic Extract for the Identification of Novel Compounds
Source: Metabolites. 2026 May 29;16(6):369. doi: 10.3390/metabo16060369 (PMC13302855; doi:10.3390/metabo16060369)
Supplement: Supplementary file 1 [file metabolites-16-00369-s001.zip › metabolites-4263925-supplementary.pdf]

Supplementary Materials

Metabolomics Analysis of Aged Garlic Extract for the Identification of Novel Compounds

Masato Nakamoto [1,2,] *, Tsubasa Nishimura [1], Masahiro Ohtani and Toshiaki Matsutomo [1]

[1] Central Research Institute, Wakunaga Pharmaceutical Co., Ltd., 1624 Shimokotachi, Koda–cho, Akitakata–shi, Hiroshima 739-1195 Japan; nakamoto_ms@wakunaga.co.jp (M.N.); nishimura_t@wakunaga.co.jp (T.N.); ootani_m@wakunaga.co.jp (M.O.); matsutomo_t@wakunaga.co.jp (T.M.)

[2] Graduate School of Integrated Sciences for Life, Hiroshima University, Higashi–Hiroshima–Shi, Hiroshima 739-8528 Japan; nakamoto_ms@wakunaga.co.jp (M.N.)

* Correspondence: nakamoto_ms@wakunaga.co.jp (M.N.); Tel.: +81 826 45 2331 (M.N.)

Correspondence

Masato Nakamoto

Central Research Institute

Wakunaga Pharmaceutical Co., Ltd.

1624 Shimokotachi, Koda–cho, Akitakata–shi

Hiroshima

Japan

Phone: +81 826 45 2331

Fax: +81 826 45 4351

nakamoto_ms@wakunaga.co.jp

Contents

Figure S1. Identification process of the components in 3rd separated fractions of AGE.

Figure S2. Effect of AGE on IL-6 production and cell viability in mouse splenic lymphocytes.

Figure S3. Chromatogram of hydrophilic sulfur compounds in aged garlic extract by post-column HPLC analysis.

Figure S4 (a)-(m). Tentative identification of the compound by LC-MS/MS.

Table S1. Characterization of hydrophilic sulfur compound in AGE from post-column HPLC chromatogram.

Figure S1. Identification process of the components in 3rd separated fractions of AGE.

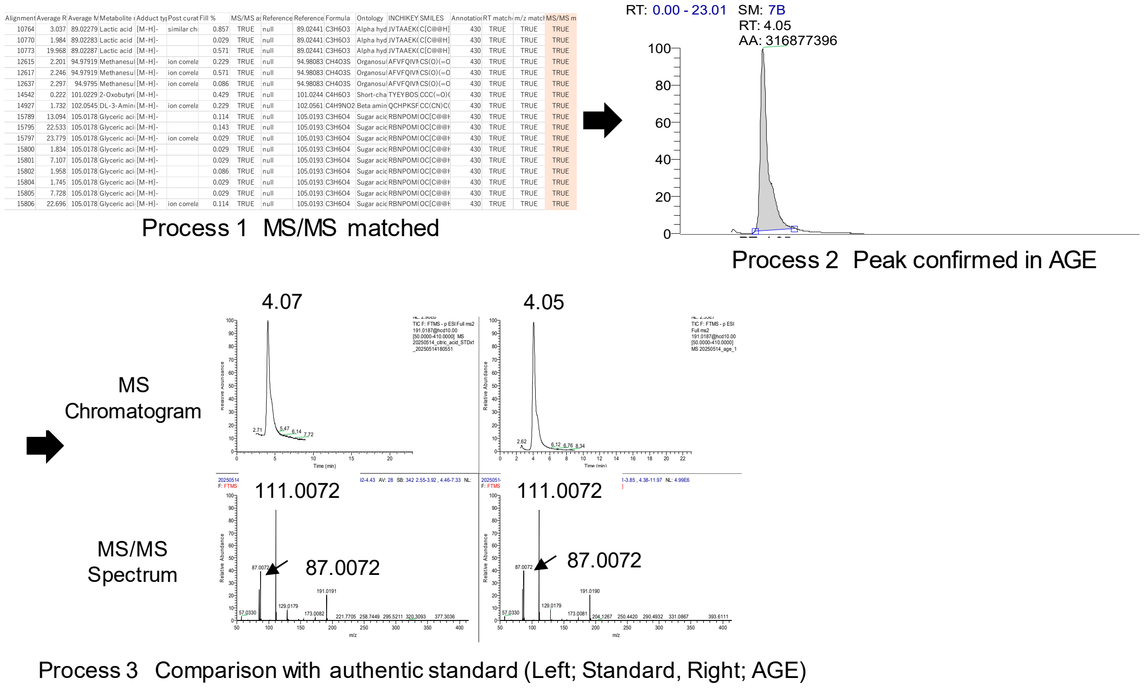

Process 1: Extraction of candidates with matched MS/MS spectra using a database search., Process 2: Confirmation of detection in AGE., Process 3: Identification by comparison with authentic standards.

Figure S2. Chromatogram of hydrophilic sulfur compounds in AGE by post-column HPLC analysis.

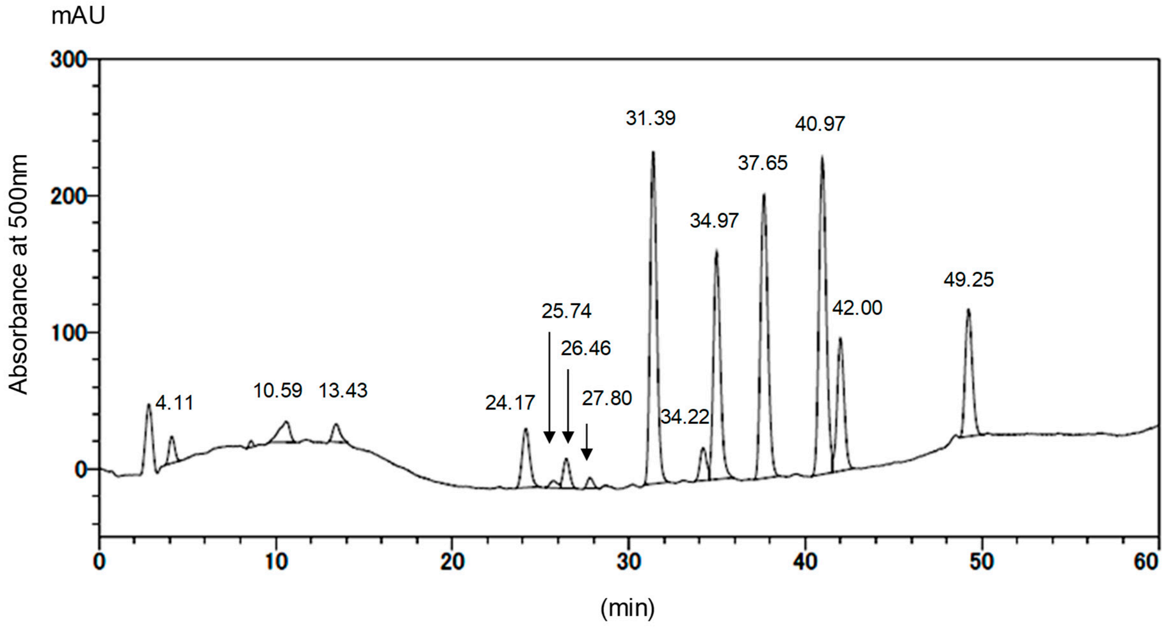

Chromatogram of hydrophilic sulfur compounds in AGE by post-column HPLC analysis. Chromatogram was monitored with 500 nm absorbance.

Figure S3. Effect of AGE on IL-6 production and cell viability in mouse splenic lymphocytes.

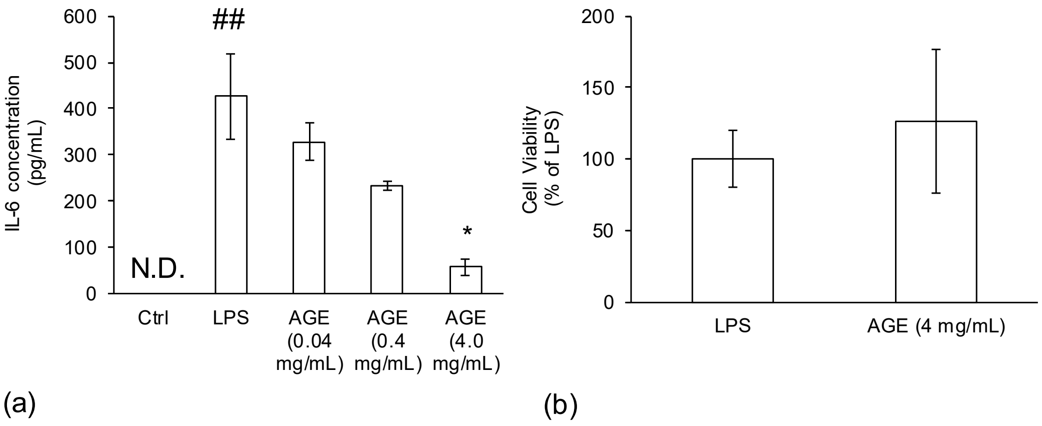

(a) Effect of AGE on IL-6 production. Splenic lymphocytes were treated with 1 μg/mL lipopolysaccharide (LPS) in the absence (control, ctrl) or presence of 0.04, 0.4, 4 mg/mL AGE for 24 h. The content of IL-6 in the culture medium was determined by ELISA. (b) Effect of AGE on cell viability. After splenic lymphocytes were simultaneously treated with LPS (1 μg/mL) and AGE (4 mg/mL) for 24 h, cell viability was evaluated by bromodeoxyuridine (BrdU) assay. Values are mean ± SD (*n*=4-5). Experiments were performed independently twice as biological replicates, and each experiment included 4-5 technical replicates. ##p < 0.01 vs control, *p < 0.05 vs LPS alone (welch-game-howell test). N.D. means not detected.

Figure S4 (a)-(m). Tentative identification of the compound by LC-MS/MS.

(a1) LC-MS Result

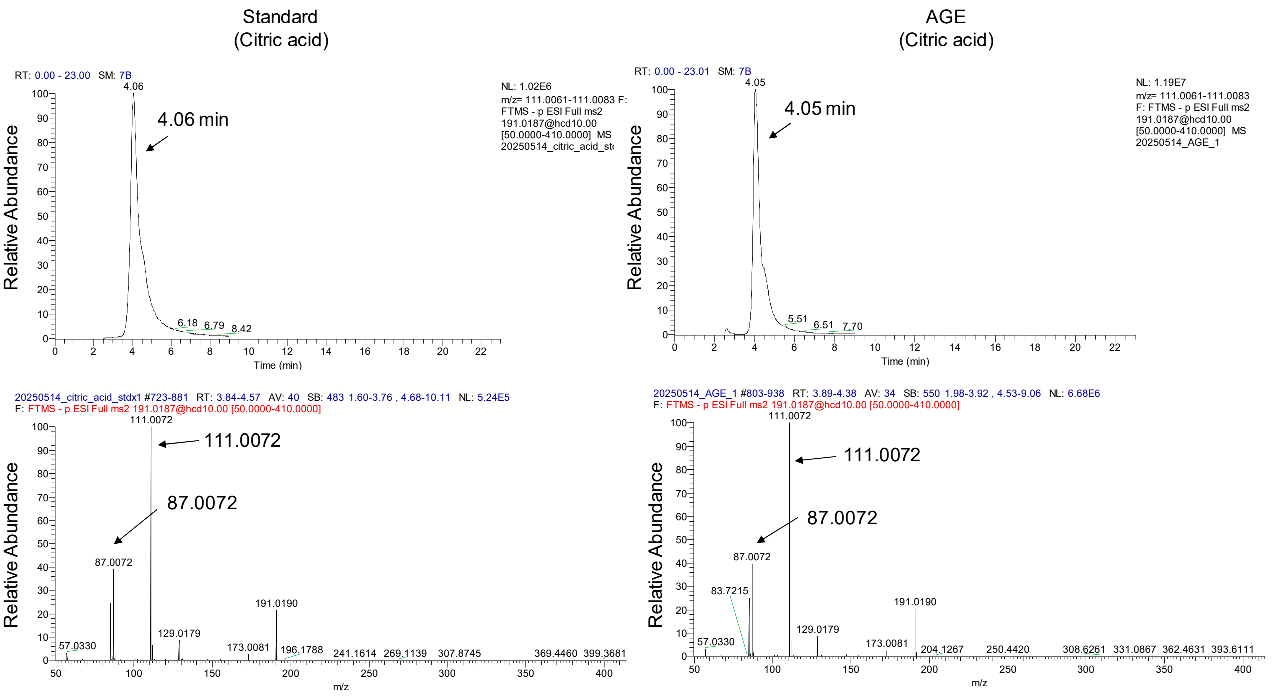

(a2) Proposed fragmentation pathway

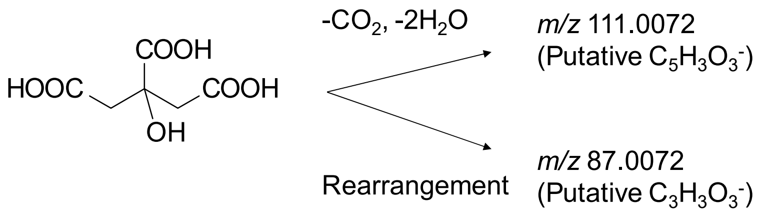

(b1) LC-MS Result

Standard (Galacturonic acid) | AGE (Galacturonic acid)

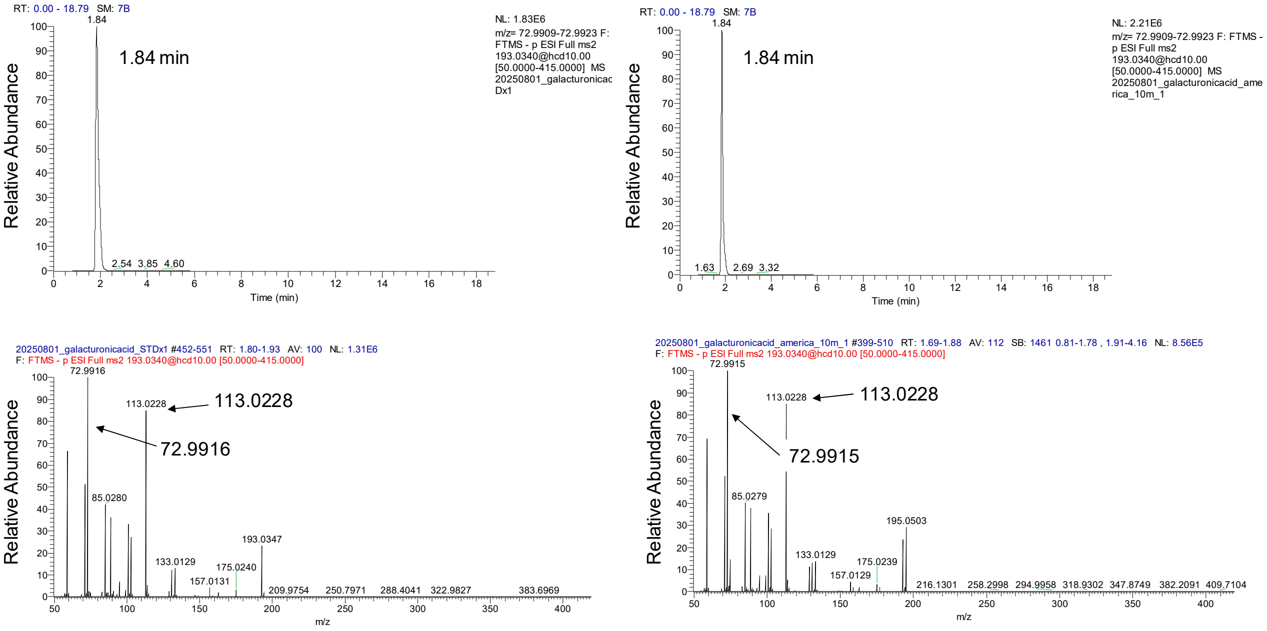

(b2) Proposed fragmentation pathway

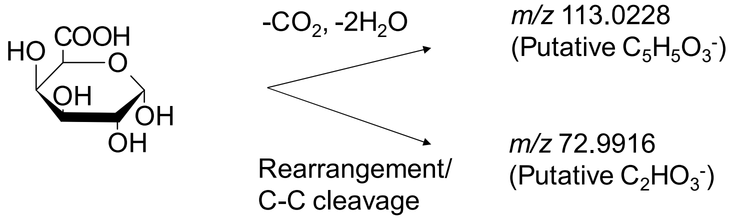

(c1) LC-MS Result

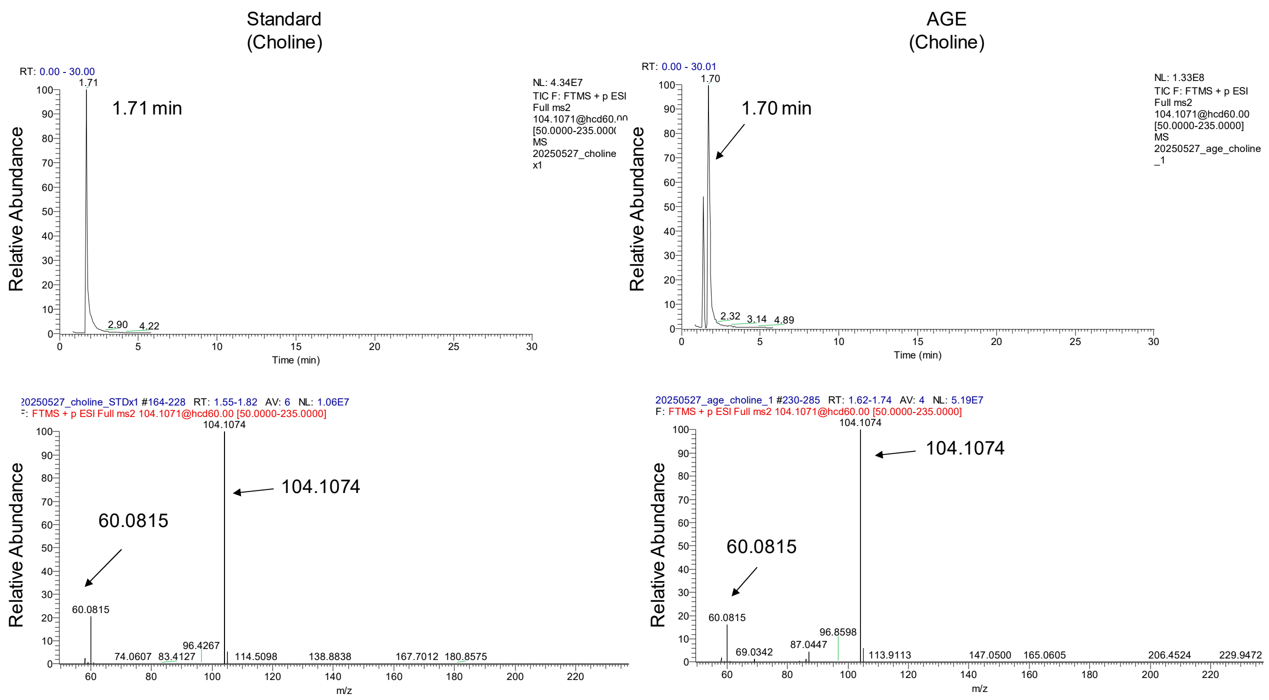

(c2) Proposed fragmentation pathway

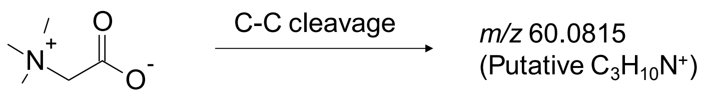

(d1) LC-MS Result

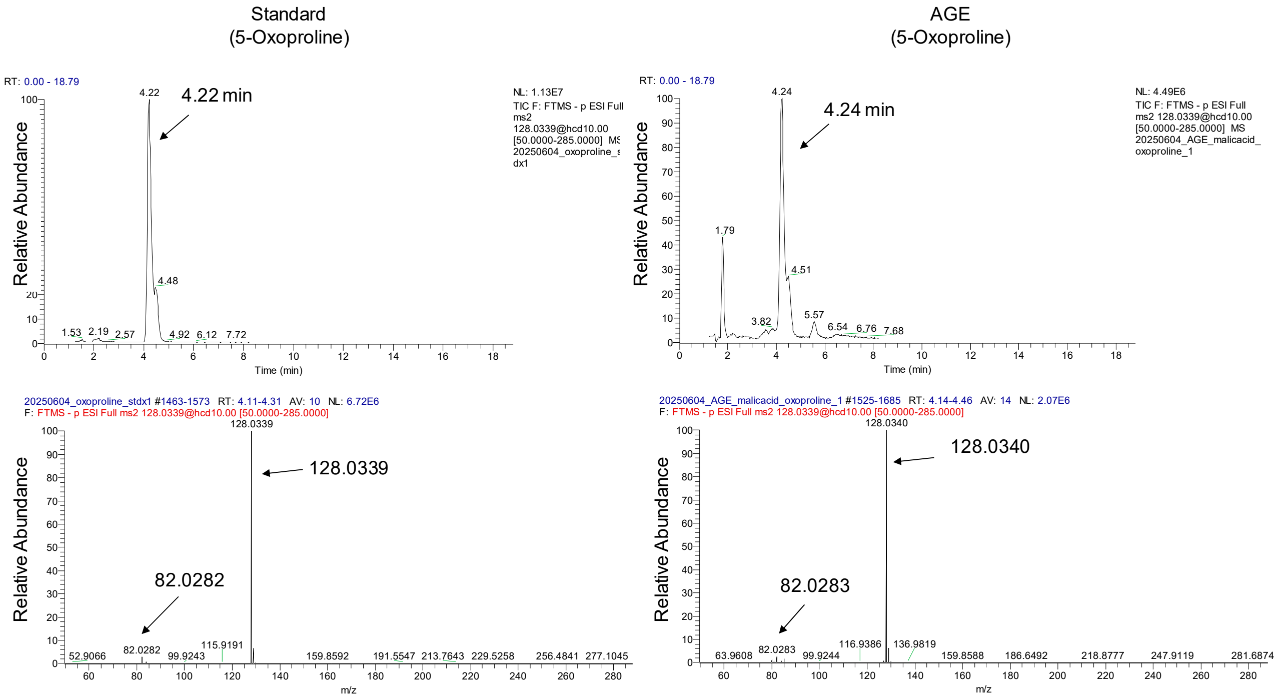

(d2) Proposed fragmentation pathway

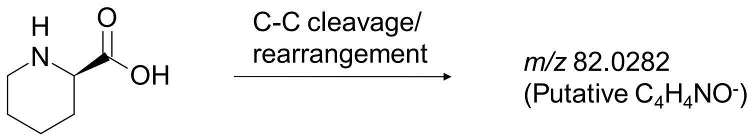

(e1) LC-MS Result

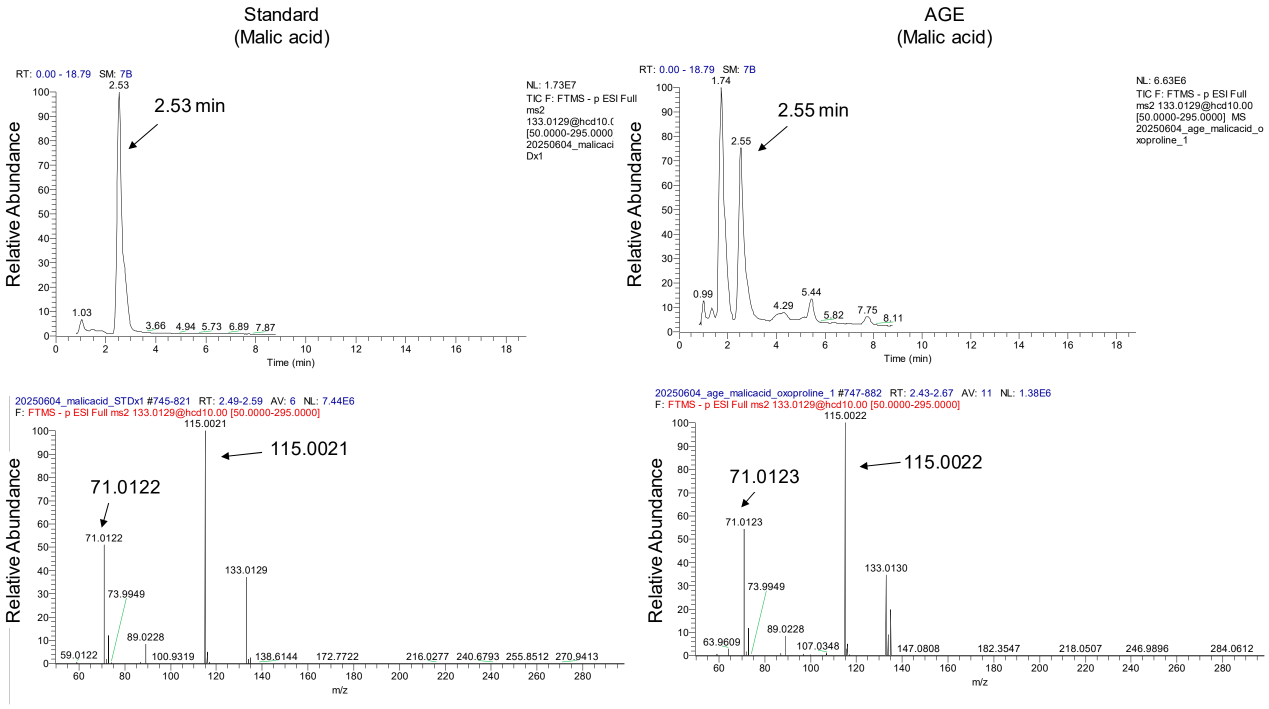

(e2) Proposed fragmentation pathway

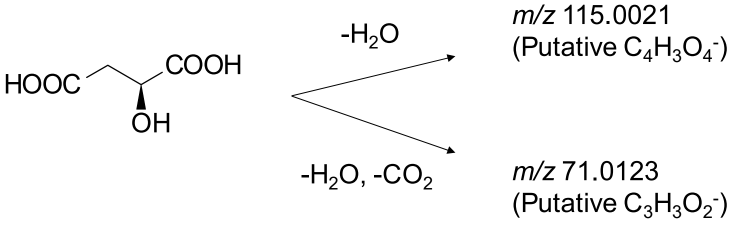

(f1) LC-MS Result

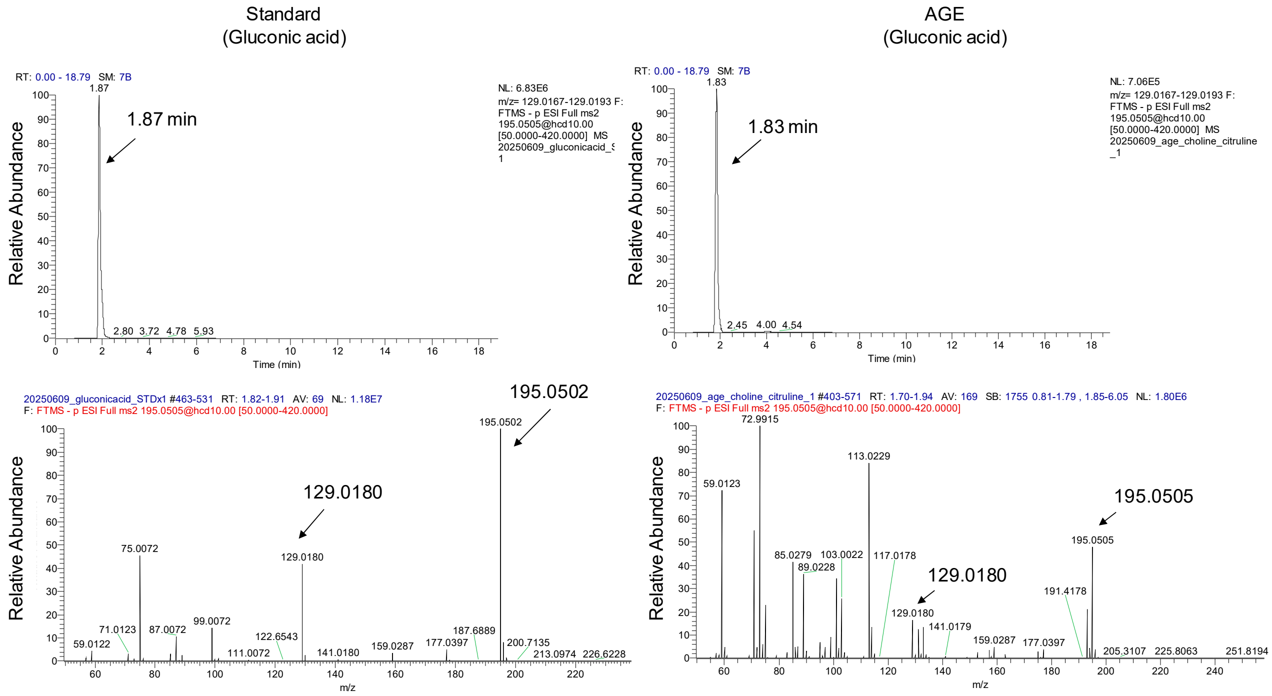

(f2) Proposed fragmentation pathway

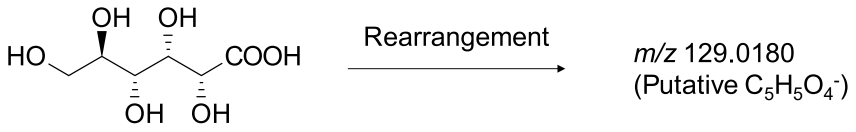

(g1) LC-MS Result

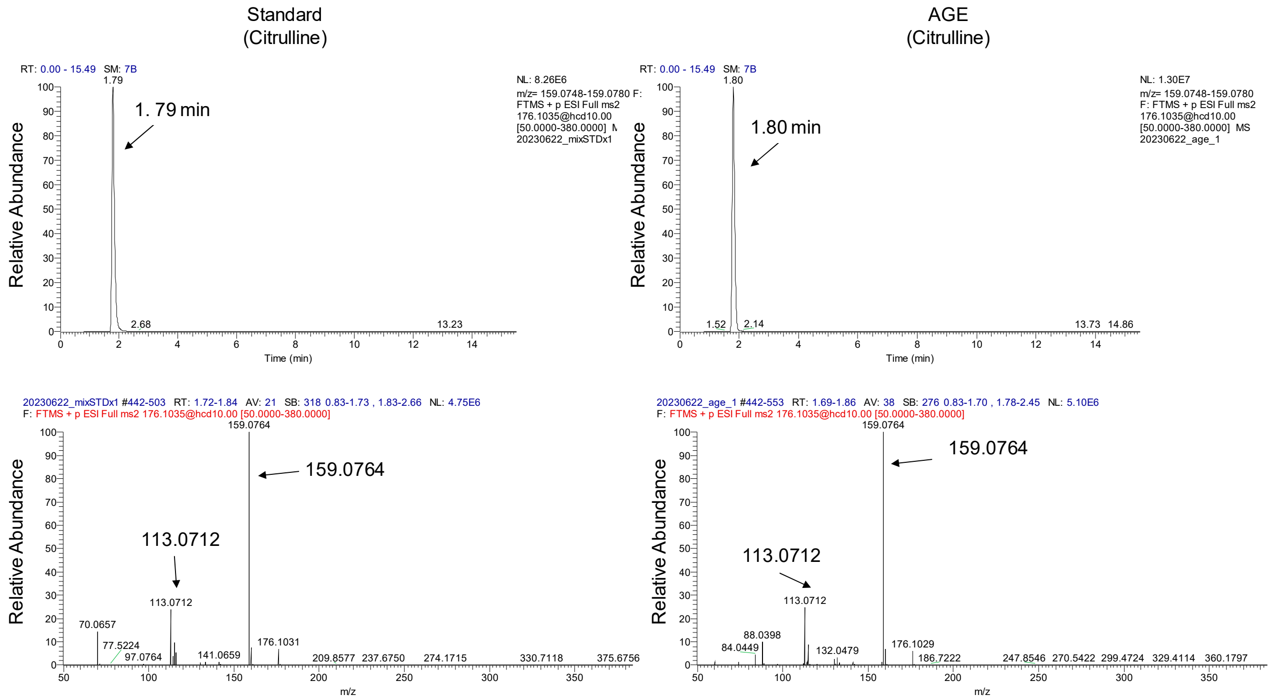

(g2) Proposed fragmentation pathway

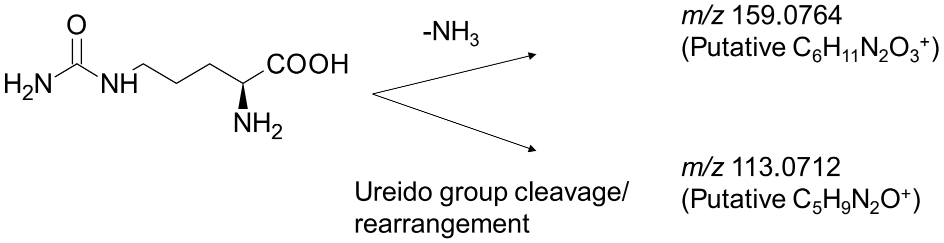

(h1) LC-MS Result

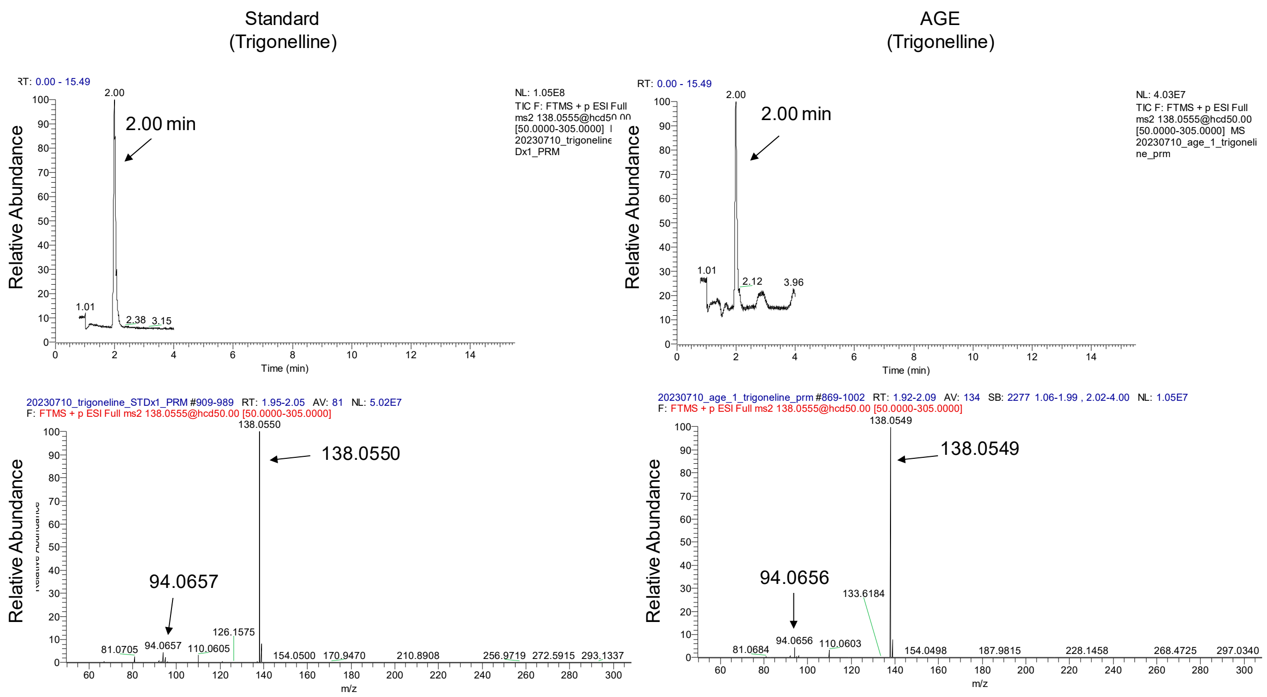

(h2) Proposed fragmentation pathway

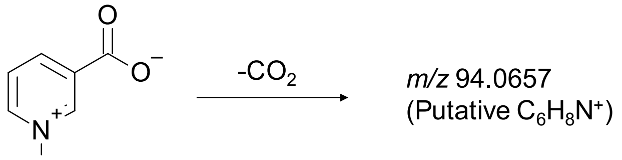

(i1) LC-MS Result

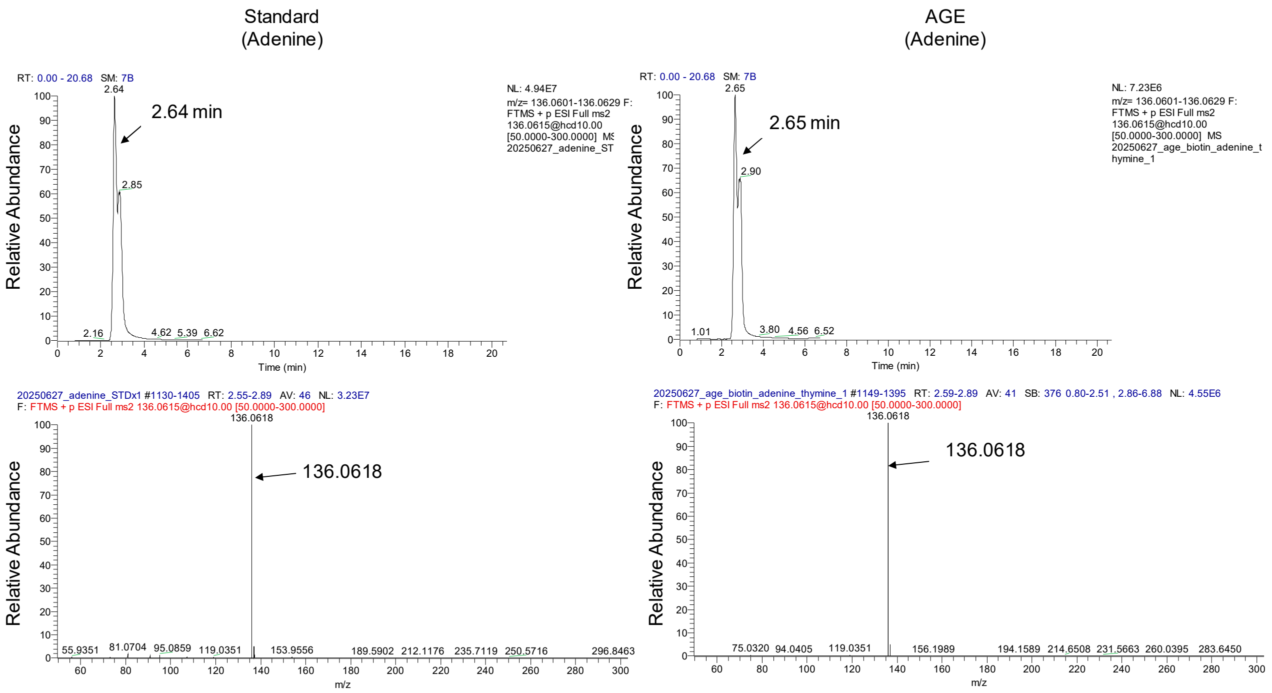

(i2) Proposed fragmentation pathway

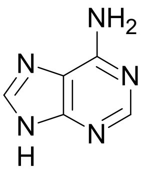

m/z 136.0618
(Only precursor ion)

(j1) LC-MS Result

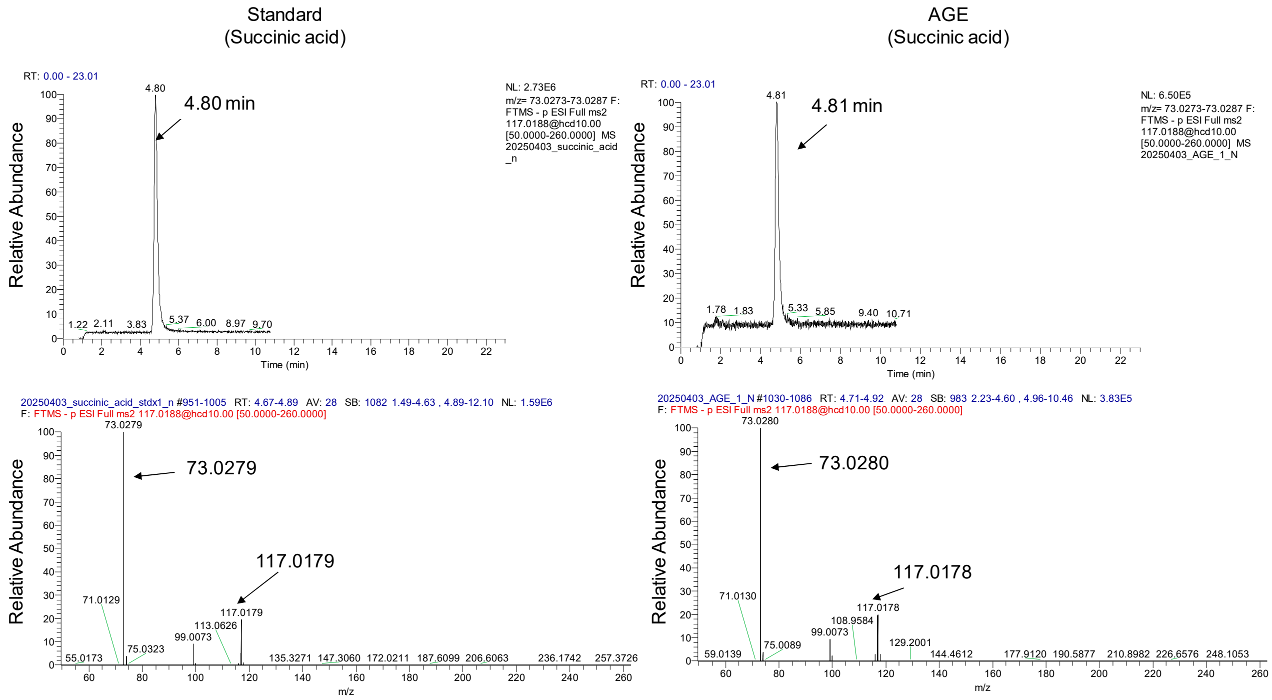

(j2) Proposed fragmentation pathway

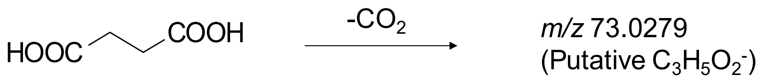

(k1) LC-MS Result

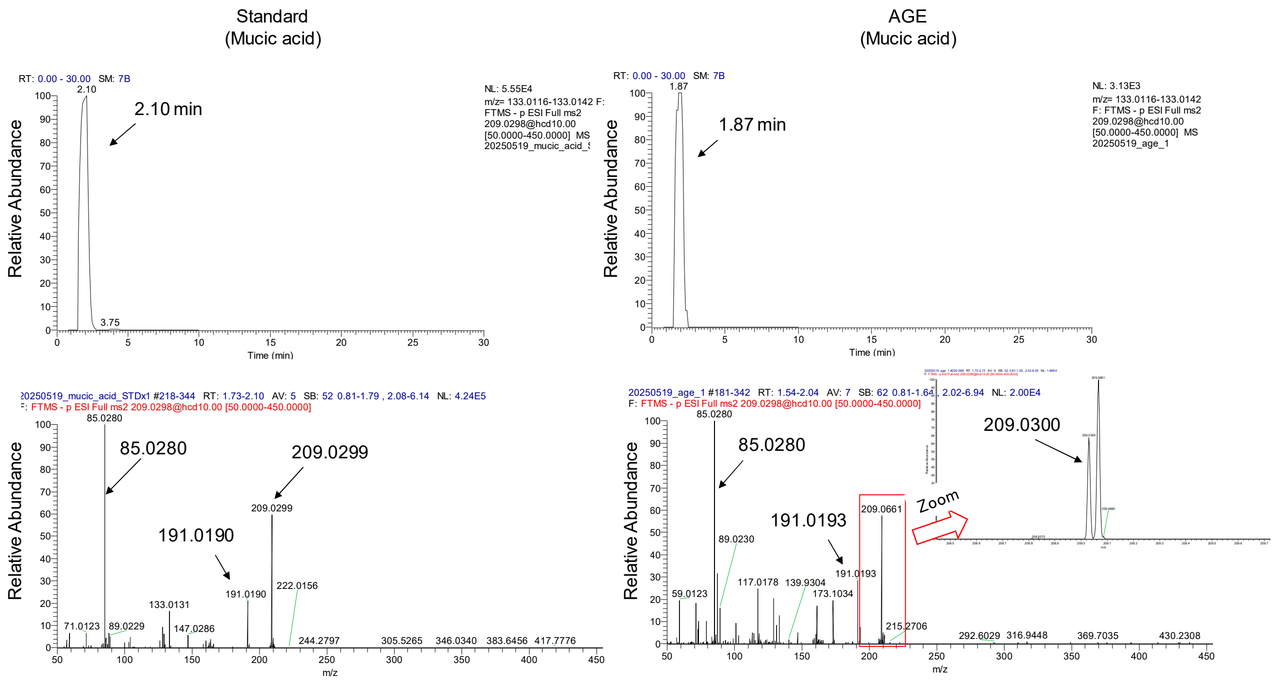

(k2) Proposed fragmentation pathway

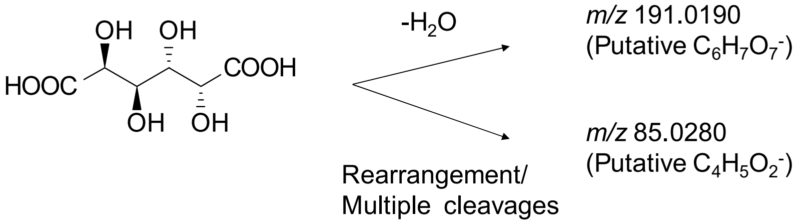

(I1) LC-MS Result

Standard (Pipecolinic acid) | AGE (Pipecolinic acid)

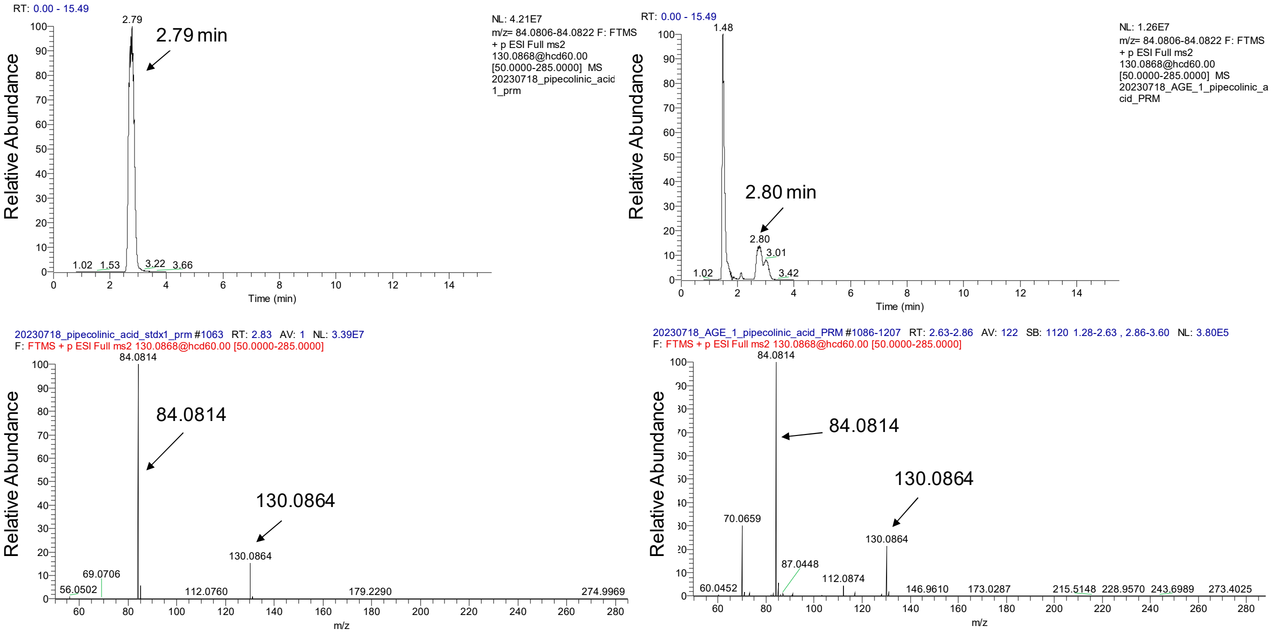

(I2) Proposed fragmentation pathway

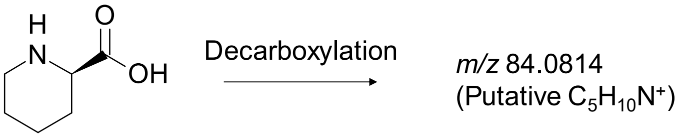

(m1) LC-MS Result

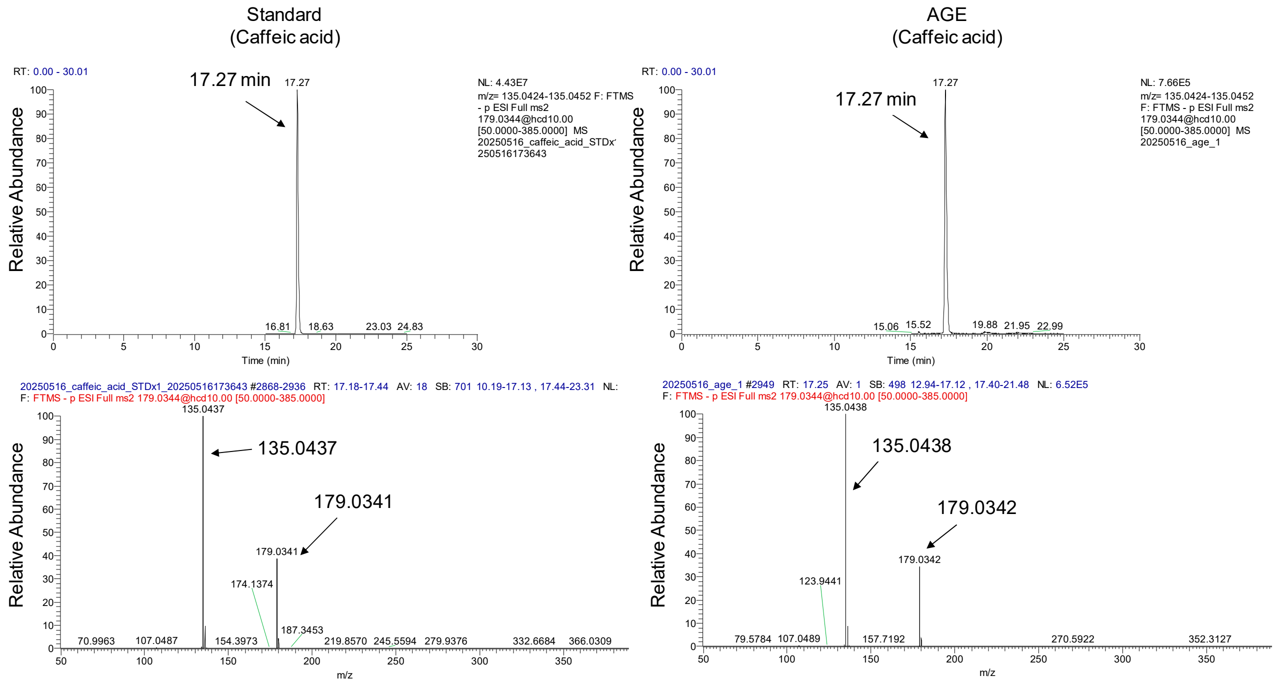

(m2) Proposed fragmentation pathway

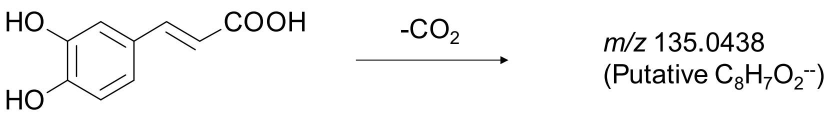

(a1-m1) The top and bottom figures show the LC-MS chromatogram and MS/MS spectrum, respectively. (a2-m2) Putative fragment ion information is shown for each compound.

Table S1. Characterization of hydrophilic sulfur compound in AGE from post-column HPLC chromatogram.

Rt	MW	Area (%)	Compounds	Contents (mg/g Dry)
24.17	149	3.88	Methionine	0.21
26.46	167	1.64	*S*-Methylmercaptocysteine	0.15
31.39	161	19.22	*S*-Allylcysteine	4.91
34.22	161	1.81	*cis*-*S*-1-propenycysteine	0.30
34.97	161	13.75	*trans*-*S*-1-propenycysteine	4.53
37.65	290	18.12	γ-Glutamyl-*S*-allylcysteine	4.33
40.97	175	20.04	*S*-n-Butenylcysteine (Internal standard)	
42.00	193	8.07	*S*-Allylmercaptocysteine	0.45
49.25	322	7.80	γ-Glutamyl-*S*-allylmercaptocysteine	0.93
